# Care leaders’ moral distress in older adult care: A scoping review

**DOI:** 10.1177/09697330251315939

**Published:** 2025-02-06

**Authors:** Fanny Ahokas, Marit Silén, Anna T. Höglund, Jessica Hemberg

**Affiliations:** 1040 Åbo Akademi University; University of Gävle; 8097 University of Uppsala; 1040 Åbo Akademi University

**Keywords:** Care leaders, care of older adults, moral distress, older adult care, scoping review

## Abstract

Moral distress among nurses is well researched and well documented, but there is limited research on the moral distress experienced by care leaders, who serve as intermediaries between patient care nurses and higher levels of administration. Healthcare professionals experience moral distress daily in the context of older adult care. The aim of this scoping review was to evaluate recent literature on moral distress in older adult care with the goal of revealing how care leaders’ experiences of moral distress in older adult care have been conceptualized in earlier studies. The research questions were: How is the concept of moral distress as experienced by care leaders in older adult care defined in the existing literature? How is the concept of moral distress conceptualized in the literature? The research has been conducted in accordance with the guidelines set forth by the Finnish National Advisory Board on Research Ethics TENK. We saw that consensus on how moral distress is defined is lacking. Care leaders in older adult care experience substantial moral distress, which could be linked to the duality of their leadership role. Moral distress can be caused by a complex interplay of individual and structural factors and the challenging complex moral issues inherent to older adult care. Moral distress could impact care leaders’ emotional health, job performance, overall job satisfaction and result in higher turnover rates, absenteeism, decreased quality of patient care, and increased organizational costs. Addressing moral distress on the individual, team, and organizational levels is crucial for enhancing care leaders’ well-being and improving the overall quality of care for older adults. A focus on the identification of strategies whereby care leaders can be supported, exploration of the long-term effects of moral distress on healthcare professionals in general, and the organizational outcomes associated with moral distress should be included in future research.

## Introduction

Moral distress among nurses is a well-researched and well-documented phenomenon, but there is limited research on the moral distress experienced by care leaders. Care leaders act as intermediaries between the nurses who provide direct patient care and those on higher administrative levels. Healthcare professionals experience moral distress daily in the context of older adult care,^1^ and researchers have previously identified moral distress as a reason underlying why many healthcare professionals in recent years have chosen to leave their professions.^2–4^ In this study, a scoping review was undertaken to evaluate recent literature on moral distress in older adult care with the goal of revealing how care leaders’ experiences of moral distress in older adult care have been conceptualized in earlier studies.

### Background

Moral distress can be defined as something that occurs when a person understands what the right thing to do is but is unable to act accordingly due to external obstacles.^5^ Moral distress can also be defined as a psychological imbalance that occurs when healthcare professionals are aware of the ethically appropriate action required but cannot perform this action due to an external obstacle,^6,7^ such as power structures, legal constraints, or lack of time.^6,8,9^

A subjective experience, moral distress affects each individual differently.^10^ While it can be considered an ethical phenomenon where ethical and moral obligations come into conflict, one can also experience moral distress without experiencing any internal conflict.^11–13^ Moral distress can lead to work-related stress, reduced self-esteem, or negative emotions, such as frustration, anxiety, sadness, or fear^2,6–9,14,15^ and also physical responses, such as loss of appetite, headaches, or fatigue.^16^ Yet moral distress can even lead to positive outcomes such as professional growth, a stronger sense of responsibility for patients, or positive emotions, and improved self-reflection.^11,14^

Healthcare professionals often find themselves in a state of moral distress or experience a sense of unease and tension because of ethical conflicts such as an inability to act in accordance with their own ethical values.^2,6,12,13,17^ Ethical conflicts have become increasingly common in older adult care owing to rapidly changing healthcare environments and an increased emphasis on efficiency.^9,17,18^ Researchers have also found that healthcare professionals working in older adult care can experience more moral distress than those working in other healthcare settings.^19^

Care leaders can experience moral distress when torn between following directives that could compromise patient care or advocating for what they believe is the right course of action.^11^ The moral distress inherent to being unable to ethically respond to such challenging situations can negatively impact care leaders’ psychological and physical well-being.^11,16^ The moral distress that care leaders experience can be related to a lack of time, patient-related issues, relative-related issues, or other ethically difficult situations.^11^ It can also arise from an imbalance between care leaders’ personal values and organizational values or other healthcare professionals’, patients’, or relatives’ values.^11,15,20^

Moral distress, as a result of ethical conflicts can arise when the top management introduces changes or issues directives that conflict with care leaders’ ethical values,^11,18,20^ and care leaders perceive that organizational responsibilities should be prioritized over their human and ethical responsibilities.^6,21^ Especially if there are limited resources or a limited budget, care leaders might be required to prioritize, which may conflict with meeting patients’ basic needs.^16,22^ The pitting of economic values against humanistic values can also lead to conflicts with care leaders’ own values.^11,18^

Value imbalances between care leaders and their staff can lead to care leaders’ moral distress.^11,20^ For example, due to staffing shortages care leaders may hire staff who lack interest in caring for older adults, thus creating a mismatch in values between those staff and the organization.^11^ Even the inability to complete administrative work can increase care leaders’ moral distress, for example, if care leaders must provide direct patient care instead of focusing on own tasks due to staffing shortages.^11^

However, while situations characterized by moral distress can be burdensome they can also promote professional growth and positive outcomes such as enhanced self-reflection.^14^ Reflection and discussion can help care leaders learn valuable lessons and enable them to develop strategies whereby future ethical challenges can be navigated.^11,23^ Moral distress reduces work performance, and while the number of older adults in need of care is rapidly increasing, the number of healthcare professionals is decreasing.^2^ There is an urgent need to find solutions to mitigate moral distress in older adult care. Although challenging, efforts on individual, team, and institutional levels are needed to reduce moral distress among healthcare professionals in general.^24^ As shown above, new and better strategies are urgently needed to address moral distress in healthcare.^25^ Knowledge about care leaders’ moral distress is necessary as this could help organizations to identify, prevent, and make it easier to manage. Doing so can improve the quality of patient care, increase care leaders’ well-being and make healthcare organizations more efficient.^22,26^

The aim of this scoping review was to evaluate recent literature on moral distress in older adult care with the goal of revealing how care leaders’ experiences of moral distress in older adult care have been conceptualized in earlier studies. The research questions were as follows: How is the concept of moral distress as experienced by care leaders in older adult care defined in the existing literature, and how is the concept of moral distress conceptualized in the literature.

## Research design

It was determined that a scoping review was the most relevant study design. A scoping review is an overview study through which the aim is to provide a picture of the research available in a field.^27,28^ Scoping reviews are considered appropriate if a systematic review design does not satisfy the study purpose.^27,29^ A scoping review also facilitates focus on a broader question and can thus be considered preferable to a systematic review if many different study designs might be applicable.^27–30^ Furthermore, a scoping review can be considered appropriate when the purpose of the study is to identify knowledge gaps, examine research conduct, or clarify concepts.^27–30^

For the purposes of this study, the scoping review framework first described by Arksey and O’Malley^29^ and further advanced by Levac, Colquhoun, and O’Brien^30^ and Peters, Godfrey, Mclnerney, Munn, Tricco, and Khalil^31^ was used. To effectively guide this scoping review, the Population, Concept, Context (PCC) framework recommended by the Joanna Briggs Institute (JBI)^31^ was used. The PCC framework facilitates the setting of clear and meaningful objectives and eligibility criteria appropriate for a scoping review. The PCC framework can be used to identify main concepts, “break down” research questions, inform search strategies, and ensure relevant and important inclusion and exclusion criteria appropriate for the study protocol. Use of the PCC framework allows for the organization of the research around the population of interest, the main concepts, and the specific context, thus improving the clarity and focus of the study and yielding a more thorough and reliable review process.^31^ The research questions were as follows: How is the concept of moral distress experienced by care leaders in older adult care as defined in the existing literature, and how is the concept of moral distress conceptualized in the literature? Seen in terms of this scoping review, the PCC framework was extrapolated as care leaders (P) experiencing moral distress (C) in older adult care (C).

The following steps were undertaken: definition of the topic; definition of the research question through use of the PCC framework; development of inclusion criteria; description of planned evidence search; identification of relevant studies; screening, organizing, and charting of the data; risk of bias appraisal; and summarization and description of the evidence so as to answer the research question.^31^

## Study selection

### Development of inclusion criteria

To gain a current and timely overview of the research topic, studies conducted and published between 2010 and 2024 were included. The start date of 2010 was chosen because since then more perspectives and research have started to emerge on this challenging topic. Peer-reviewed and gray literature (theses, white papers, policy statements, and conference reports) were included, as well as studies in all languages to get a more reliable result.^28,31^ Also included were studies in which moral distress was defined, and studies in which moral distress was experienced by care leaders in older adult care.

### Description of planned evidence search

The search for evidence was planned to encompass the identification of relevant studies via searches of selected databases, with the goal of retrieving all that matched the search criteria, that is, the inclusion and exclusion of studies in accordance with stated inclusion and exclusion criteria. The search included an initial review of all titles and abstracts followed by the obtaining and assessment of the full text, in line with the inclusion and exclusion criteria. Each article was then thoroughly read to determine its relevance to the stated aim of the study.

### Identification of relevant studies

**Figure 1.**
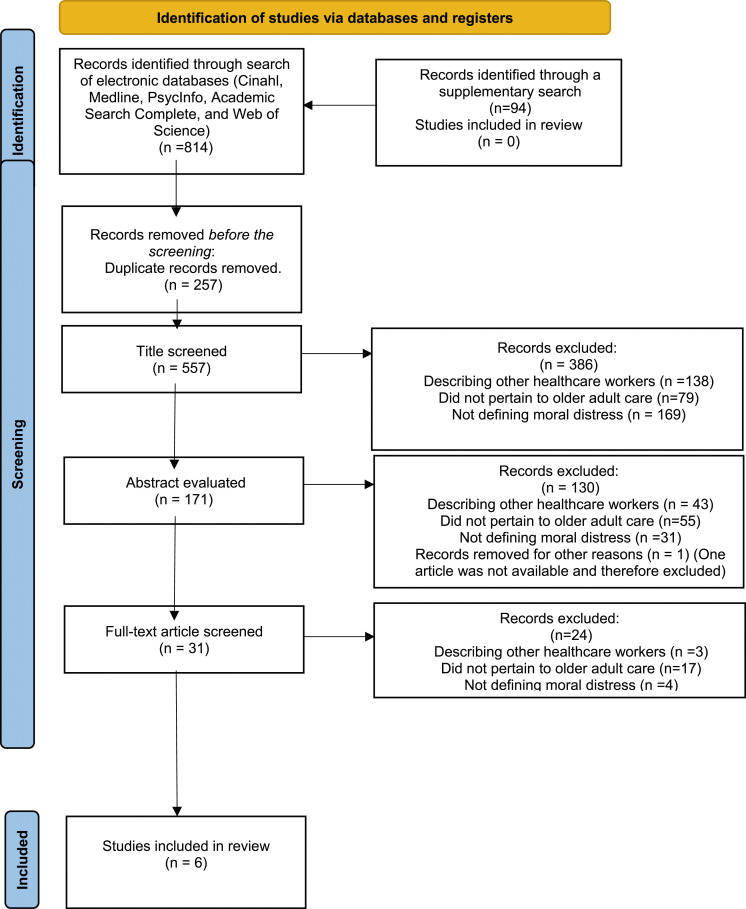
Preferred Reporting Items for Systematic Reviews and Meta-Analyses Extension for Scoping Reviews (PRISMA-ScR) flow chart of the study selection.

A literature search was conducted in accordance with Preferred Reporting Items for Systematic Reviews and Meta-Analyses Extension for Scoping Reviews (PRISMA-ScR) guidelines.^32^ The databases and registers searched included Cinahl, Medline, PsycInfo, Academic Search Complete, and Web of Science. For an overview of this process, see Figure 1.

Designed with help from a librarian, the Medical Subject Headings (MeSH) terms ethics and nursing were chosen as key search terms for the data search process Table 1. These terms were combined with various synonyms for distress, stress, and older adult care using Boolean operators (AND, OR) and appropriate truncation and wildcard symbols, so as to capture variations. A supplementary search of all databases was carried out on June 4, 2024, to ensure that all relevant data had been included in the scoping review. New hits were found in all databases (*n* = 94), but after scanning none (*n* = 0) of these studies were determined to have met the study inclusion criteria. In total, six studies were included in the scoping review. For an overview of the data search process, see Table 2.

**Table 1.** Inclusion and exclusion criteria.

Inclusion criteria	Exclusion criteria
Published between 2010 and 2024	Published before 2010
Peer-reviewed and gray literature (theses, white papers, policy statements, and conference reports)	Inaccessible DOI
All languages	
Qualitative, quantitative, or mixed-method studies	Not available in full text
Definition of moral distress	No definition of moral distress
Moral distress experienced by care leaders in older adult care	Moral distress experienced by other healthcare workers or other contexts

**Table 2.** Overview of the database search process.

Database Date	Search words	Hits
Academic search complete	Moral distress OR ethical dilemma OR moral stress OR ethical issues OR ethical stress OR ethical distress OR psychological distress OR stress, Physiological OR Occupational stress OR stress	102
	26.9.2023	
	AND	
	Ethics nursing	
	AND	
	Aged care OR nursing home OR residential aged care facility OR long-term care OR aged OR elderly OR senior OR older people	
CINAHL	Moral distress OR ethical dilemma OR moral stress OR ethical issues OR ethical stress OR ethical distress OR psychological distress OR stress, Physiological OR Occupational stress OR stress	78
	26.9.2023	
	AND	
	Ethics nursing	
	AND	
	Aged care OR nursing home OR residential aged care facility OR long-term care OR aged OR elderly OR senior OR older people	
MEDLINE	Moral distress OR ethical dilemma OR moral stress OR ethical issues OR ethical stress OR ethical distress OR psychological distress OR stress, Physiological OR Occupational stress OR stress	223
	26.9.2023	
	AND	
	Ethics nursing	
	AND	
	Aged care OR nursing home OR residential aged care facility OR long-term care OR aged OR elderly OR senior OR older people	
PsycInfo	Moral distress OR ethical dilemma OR moral stress OR ethical issues OR ethical stress OR ethical distress OR psychological distress OR stress, Physiological OR Occupational stress OR stress	250
	26.9.2023	
	AND	
	Ethics nursing	
	AND	
	Aged care OR nursing home OR residential aged care facility OR long-term care OR aged OR elderly OR senior OR older people	
Web of Science	Moral distress OR ethical dilemma OR moral stress OR ethical issues OR ethical stress OR ethical distress OR psychological distress OR stress, Physiological OR Occupational stress OR stress	161
	26.9.2023	
	AND	
	Ethics nursing	
	AND	
	Aged care OR nursing home OR residential aged care facility OR long-term care OR aged OR elderly OR senior OR older people	

### Charting of the data

The full-text studies identified as appropriate for inclusion (*n* = 6) were synthesized and summarized to answer the research question. Studies that encompassed care leaders; described moral distress; used qualitative, quantitative, or mixed methodology; were peer-reviewed research; or were gray literature were included. Excluded from the records were studies that focused on other healthcare workers; did not pertain to older adult care; did not address ethics, ethical stress, or ethical dilemmas; or did not define moral distress. Three studies included both care leaders and nurses in their sample^33–35^ but were included because they were considered to appropriately describe the experience of care leaders. For an overview of the studies included in the scoping review (an asterisk for these studies is indicated in the reference list), see Table 3.

**Table 3.** Overview of the studies included in the scoping review.

Author/Year	Country	Aim	Design	Sample/Setting	Main results	Definition of moral distress/Source for definition
Atli Özbas & Kovanci 2022	Turkey	To explore the moral distress experiences of nurse officers during the COVID-19 pandemic	A descriptive phenomenological study and content analysis	(*n* = 13) 13 chief/assistant nurse officers	Confronted with multiple demands, including handling unpredictable and unfamiliar processes, as well as addressing the psychological reactions of both their staff and themselves, chief nurse officers faced considerable challenges in preserving their moral integrity and experienced moral distress during the COVID-19 pandemic	*Moral distress (MD) was first defined by Jameton in 1984 as* “*the distress experienced by a person when, despite knowing the right action to take, it is almost impossible to follow the right course of action due to institutional constraints*” (p. 2) Corley, 2002 Jameton, 2017
de Veer et al., 2013	Netherlands	To identify individual and job characteristics associated with moral distress in Nursing staff	Cross-sectional correlational study	(*n* = 365)	Higher levels of moral distress were associated with reduced job satisfaction among nurses. Moral distress tended to increase when nurses felt they had insufficient time to provide proper care to patients	*Moral distress consists of negative stress symptoms that occur in situations that involve ethical dimensions and where the nurse feels (s)he is not able to preserve all interests and values at stake. …moral distress is the painful psychological disequilibrium that results from recognising an ethically appropriate action that is difficult to take…* (p. 111)
				Nursing staff members employed in Nursing homes, homes for the elderly, home-care and acute-care hospitals 9% worked with management tasks	Additionally, low satisfaction with team consultations and the presence of an instrumental leadership style contributed to greater moral distress. Nurses who worked 30–40 hours per week experienced less moral distress compared to those working fewer hours per week	Kälvemark Sporrong, Höglund and Arnetz, 2004 Corley, Elswick, Gorman and Clor, 2001
Jakobsen et al. 2023	Norway	To explore healthcare leaders’ expectations of using a digital tool for ethical	Qualitative research design Vignettes and focus group interviews	(*n* = 10) Home nursing care	Leaders need to be aware of the ethical challenges their staff face to provide effective support	*Moral distress refers to the psychological stress experienced as a consequence of encountering moral*
						
		reflection among their home nursing care staff	Reflexive thematic analysis	Had worked as leaders for 0.5–8 years	Digital channels can enhance communication between employees and leaders by making it more democratic, secure, and efficient, while also raising awareness of ethical issues in daily care work. Leaders had positive expectations regarding the use of a tool for ethical reflection, believing it would benefit employees. However, they acknowledged that fully utilizing the tool’s potential to maintain continuity in ethics work would demand significant commitment from both the organization and its leaders	*situations characterized by moral tension, moral conflict, moral dilemma, moral uncertainty* (p. 2) Morley, Bradbury-Jones and Ives, 2020
Mitton et al. 2011	Canada	To determine whether the concept of moral distress is a	Qualitative research design	(*n* = 18)	Mid- and senior-level managers both seem to experience moral distress, though the	*Moral distress is the suffering experienced as a result of situations in which individuals feel*
		Significant issue for managers	Interviews and focus groups with mid- and senior-level managers Thematic content analysis	Mid-level managers and senior executives in two health authorities	way it manifests can vary between these groups. Examples of this include the need to communicate or “sell” organizational decisions or policies that a manager may personally disagree with, as well as situations where limited resources force managers to place staff in circumstances where they face	*morally responsible and have determined the ethically right action to take, yet due to constraints (real or perceived) cannot carry out this action, thus committing a moral offence* (p. 108) Jameton, 1984 Nathaniel, 2002 Rodney, Brown, & Liaschenko, 2004 Rushton, 2006
					foreseeable and potentially preventable risks	
						
Petersen & Melzer, 2023	Germany	To explore the phenomenon of moral distress and describe its work-related predictors and individual consequences for home-care nurses in Germany	Cross-sectional design Moral distress scale and COPSOQ III questionnaire within the framework of an online survey Frequency analyses, multiple linear, logistic regressions	(*n* = 976) Home-care nurses 50.1% in management positions	Participants experienced moral distress when they were unable to prevent patient suffering due to factors like inadequate physician orders, being required to follow orders for tests or treatments they deemed inappropriate, or	*When nurses cannot act according to their ethical beliefs, they may experience moral distress…Jameton…stated that [moral distress] arises* “*when one knows the right thing to do, but institutional constraints make it nearly impossible to pursue the right course of action*” (p. 2)
			Rasch analyses		having to work with colleagues they considered incompetent	Jameton, 1984
					Safety was framed as a conflict between “welfare versus loyalty” and “welfare versus autonomy.” Poor communication and working with unqualified colleagues also contributed to moral distress	
					Younger nurses, those with less professional experience, and female nurses reported higher levels of moral distress. Nurses working in patients' homes experienced greater moral distress due to limited flexibility in their roles	
					Moral distress was linked to lower health	
					outcomes, and job and organizational factors influenced the extent of distress experienced	
Weiste et al., 2023	Finland	To study the ways in which elderly care practitioners discuss moral distress in their work	Combination of qualitative methods deriving from interaction-oriented focus group research, conversation analysis and discursive psychology Analytic transcription conventions	(*n* = 38) Single unit supervisor (*n* = 3) and 9–13 care workers (*n* = 35) Two workshops in three different elderly care units (two service housing subunits and a geriatric ward)	Most instances of moral distress were connected to teamwork, particularly concerning elderly care practitioners' ability to influence their work and collaborate with supervisors and colleagues. The principle of equal treatment of clients is a fundamental norm, and violations of this principle in client care were experienced as distressing. Additionally, difficulties in collaborative relationships contributed to moral distress, especially when practitioners' views on what constitutes good care differed from those of their clients	*The term moral distress has been used to refer to situations in which a person is constrained from acting on what they think is right or in which they are uncertain of the right solution to the moral problem* (p. 2) Fourie, 2015

### Ethical considerations

The research has been conducted in accordance with the guidelines set forth by the Finnish National Advisory Board on Research Ethics TENK^36^. This scoping review is the second sub-study in a PhD research project, with the purpose to examine the experiences of care leaders and nurses regarding moral distress in older adult care, in order to gain an understanding of the phenomenon and explore ways to reduce moral distress. Ethical approval was granted for the PhD research project by an ethical committee at a setting where the researchers are domiciled. Ethical approval was not required for this scoping review.

## Findings

The aim of this scoping review was to evaluate recent literature on moral distress in older adult care with the goal of revealing how care leaders’ experiences of moral distress in older adult care have been conceptualized in earlier studies. The research questions were as follows: How is the concept of moral distress as experienced by care leaders in older adult care defined in the existing literature, and how is the concept of moral distress conceptualized in the literature.

### Definition and conceptualization of moral distress

How moral distress was defined and conceptualized varied widely between the various included studies. Of the studies, Atli Özbas and Kovanci^37^ defined moral distress in accordance with definitions set forth by Corley^38^ and Jameton.^39^ De Veer et al.^33^ defined moral distress in accordance with Kälvemark Sporrong, Höglund and Arnetz^40^ and Corley et al.^38^ Jakobsen et al.^41^ defined moral distress in accordance with Morley, Bradbury-Jones, and Ives.^42^ Mitton et al.^43^ defined moral distress in accordance with Jameton,^5^ Nathaniel,^44^ Rodney, Brown, and Liaschenko,^45^ and Rushton.^46^ Petersen and Melzer^34^ defined moral distress in accordance with Jameton.^5^ Weiste et al.^35^ defined moral distress in accordance with Fourie.^12^ Although definitions vary, they still have the same meaning and are rooted in Jametons’ definition: distress is experienced by a person when it is almost impossible to follow the right course of action due to institutional constraints, despite knowing the right action to take. The variations of the definitions are displayed in Table 3.

In all of the studies, moral distress was found to be characterized as negative stress symptoms that can arise when an individual recognizes the ethically appropriate action to take but feels unable to act upon said action due to different constraints.^31,33,35,37,41,43,47^ Negative stress was seen to be rooted in moral tension, conflict, dilemma, limitation, and uncertainty, while constraints could be institutional, organizational, or situational. Both negative stress and constraints were found to create a moral conflict that resulted in psychological stress.

In one study, moral distress was seen to arise from situations where an individual is not able to preserve all of the values and interests at stake.^33^ In another study, moral distress was found to result from that care leaders must both reflect on own moral and ethical values and others’ values and choices.^35^ In one study, older adult care practitioners were found to experience more moral distress than those in other sectors, which was linked to the simultaneous decrease in the number of nursing professionals and increase in the elderly population and need for care.^35^

### Moral distress among care leaders in older adult care

In one study, care leaders in older adult care were seen to face unique challenges concerning moral distress due to that they must navigate own ethical values while also supporting their staff^35^ and aligning with organizational directives.^43^ In another study, care leaders were found to often experience higher levels of moral distress compared to frontline nurses, which was attributed to care leaders’ dual responsibilities.^33^ In one study, care leaders were found to not only need to manage and be aware of own moral difficulties and distress but also that of the institution and their workers.^37^ In another study, care leaders were found to experience pressure to be role models in ethical situations and when undertaking ethical decisions.^41^

In one study, older adult care practitioners were found to experience more moral distress than those working in other sectors, which was linked to factors specific to their work environment and patient demographics. These factors were seen to include the unique ethical challenges inherent to older adult care, organizational and resource constraints, interpersonal dynamics, and the high emotional toll of the work. Alongside observations that the care of older adult patients is charged with complex ethical issues, such as the use of coercive methods, lack of informed consent, and the need to manage differing opinions between patients and their families, care leaders were seen in this study to often encounter situations where their ethical and professional responsibilities clash, which in turn was seen to create a significant source of moral distress.^35^

### Causes of moral distress in older adult care

In one study, care leaders’ moral distress was found to be caused by a complicated interaction between individual and structural factors.^33^ The individual factors comprised personal values, moral sensitivity, and ethical awareness, while the structural factors comprised organizational constraints, inadequate staffing, high job pressure, and conflicting interests between patient care and administrative directives.

Conflicts between organizational factors and patient needs were seen to be linked to care leaders’ moral distress in five studies.^33,35,37,43^ Ethical dilemmas relevant to patient autonomy and end-of-life care were found to be linked to moral distress in two studies.^33,37^ In four studies, care leaders’ moral distress was associated with discrepancies between patient, doctor or family wishes or when doctors/physicians act in a manner not aligned with care leaders’ own ethical values.^33–35,47^ In one study, moral distress was linked to the performance of managerial functions, for example, when care leaders must communicate organizational information that they do not support.^43^ In four studies, the observation of colleagues’ unethical behavior or mistakes were seen to be linked to care leaders’ moral distress,^33,34,37,41^ while having inadequate resources or the time to provide proper care were found to be linked to care leaders’ moral distress in five studies.^33–35,37,43^ In one study, part-time workers were found to have a higher moral distress level than full-time workers, which might be linked to full-time workers having more knowledge and experience in ethics and being more capable of dealing with situations that can lead to moral distress.^33^ In one study, the lack of opportunity and space to express ethical concerns and reflection was seen to negatively impact workers, care, and the environment.^41^ In two studies, a lack of support from upper leadership, including the need to perform actions that care leaders do not support, was found to be linked to care leaders’ moral distress.^35,48^ In two studies, care leaders’ primary source of moral distress was found to be linked to challenges associated with the care leader (leadership) role and organizational constraints.^33,37^

In one study, moral distress was seen to be associated with care leaders’ perceiving a lack of understanding from higher managers and that care leaders, as middle managers, experience first-hand the issues and consequences of organizational decisions, such as budgetary decisions.^43^ In one study, care leaders’ moral distress was seen to be associated with a lack of empowerment, for example, when management rendered care leaders’ decisions invalid.^37^

### Effects of moral distress in older adult care

#### The individual level

Care leaders’ moral distress was found to be linked to various negative emotions, such as anger, frustration, guilt, sadness, or powerlessness.^34,35,41,43^ It was also seen to be associated with significant impacts on psychological and physical well-being,^34,35,37^ such as burnout, depression, anxiety, headaches, or sleep disturbances.^34,35,37^ Severe cases of moral distress might result in hospitalization,^48^ and moral distress can lead care leaders to quit their jobs.^34,35^

#### The organizational level

Care leaders’ moral distress was seen to be associated with lower job satisfaction, increased turnover rates,^33,34^ and higher absences due to sick leave,^33,34,43^ which can impact the quality of patient care^41^ and increase organizational costs. Care leaders’ moral distress was even found to be linked to lower team morale,^43^ a negative work environment,^41^ and becoming more sensitive to future moral issues.^35^ Care leaders’ moral distress was furthermore seen to be associated with the intent to leave a management position, which was linked to long work hours, heavy workload, and inadequacies.^37^

### Reducing care leaders’ moral distress in older adult care

The fostering of an open and supportive culture where ethical concerns can be freely discussed was seen to possibly mitigate care leaders’ moral distress,^41^ likewise the promotion of teamwork and effective communication,^33,35,37^ and having more and broader discussions about moral dilemmas within a unit.^35^ Ensuring adequate staffing to reduce time pressure,^33^ the provision of ethics consultations and support programs,^48^ and support in the form of guidelines and scientific information^37^ were also seen to be important in the reduction of care leaders’ moral distress. The implementation of leadership styles through which support and relationship-building are emphasized over task-focused management, that is, a supportive culture, was also found to be important.^33,35^ Furthermore, the equal treatment of all parties, staff, patients, and family, was seen to be key to the reduction of care leaders’ moral distress.^35^

### Care leaders’ role in addressing and reducing moral distress in older adult care

In three studies, care leaders were seen to play an important role in addressing and reducing moral distress in older adult care, and a supportive leadership style in which care work errors are emphasized in such a manner so as to facilitate learning rather than criticism was found to lead to better communication and reduced moral distress.^33,35,41^ Care leaders’ encouragement of ethical reflection and discussions between staff, seen as the emotional and professional support of one’s team, was even found to help address and reduce moral distress.^33,35^ Also, care leaders empowering staff to act in accordance with their ethical beliefs^33,35^ and assisting in developing competence in moral and ethical situations^41^ were seen to help mitigate moral distress in older adult care. In one study, care leaders as role models for ethical decision-making and resilience in moral and ethical dilemmas was seen to be linked to reduced moral distress,^41^ while in another study seeking support and discussing moral dilemmas within the unit were found to be associated with reduced moral distress.^35^

## Discussion

The aim of the study was to evaluate recent literature on moral distress in older adult care with the goal of revealing how care leaders’ experiences of moral distress in older adult care have been conceptualized in earlier studies. We discerned that moral distress is a significant and universal issue in older adult care, driven by the conflict between ethical obligations and external constraints. We found that care leaders in older adult care face significant challenges when navigating ethical dilemmas, including the balancing of organizational directives with patient care needs and the management of moral tensions arising from such conflicts.

We saw that consensus on how moral distress is defined within the parameters specific to this review is lacking. Of the seven included studies, definitions of moral distress emanating from Jameton were most frequently used; Jameton’s 1984 definition was used in two studies and Jameton’s 2017 definition was used in one study. Improved clarity on the issue, including consensus on a standardized definition of moral distress and the use of theoretical frameworks, should be investigated in future research.

We found that care leaders in older adult care experience substantial moral distress, often more than their counterparts in other healthcare sectors. We discerned that care leaders’ moral distress could be linked to the duality of their leadership role; they must balance adhering to organizational policies that may conflict with own ethical values while also supporting their staff. We saw that moral distress among care leaders in older adult care can be caused by a complex interplay of individual and structural factors and the challenging complex moral issues inherent to older adult care. We also found that the effects of moral distress in the context of older adult care can lead to negative consequences on both the individual and organizational levels. We discerned that moral distress could impact care leaders’ emotional health, job performance, and overall job satisfaction and that moral distress can result in higher turnover rates, absenteeism, decreased quality of patient care, and increased organizational costs.

Moral distress is a pervasive problem in the care of older people. Although its effects are well documented among frontline nurses, the unique challenges faced by care leaders deserve greater attention. By addressing moral distress at the individual, team and organizational levels, and by addressing the root causes of moral distress, organizations can increase the well-being of both leaders and their teams as well as improve the overall quality of care for older people.

## Strengths and limitations

The trustworthiness of the present study was ensured by a complementary search of all databases prior to the submission of the study. One of its strengths is the inclusion of both qualitative and quantitative studies, leading to a detailed and broader picture of moral distress. Another is that no gray literature was used, owing to a lack of such and that all languages were included in the search. One limitation may be the small number of studies included in the analysis because of the dearth of studies on care leaders and moral distress in older adult care which is why the findings and comparisons can be considered somewhat limited.

## Conclusions

Moral distress is an important and common issue in older adult care, driven by the conflict between ethical obligations and external constraints. This review highlights the significant challenges care leaders face in navigating ethical dilemmas, balancing organizational directives with patients’ care needs, and managing the moral tensions that arise from these conflicts. The literature shows that there is no consensus on the definition of moral distress. Care leaders in older adult care experience significant moral distress, often more than their colleagues in the same positions in other health sectors. There is a need to develop standardized definitions and effective interventions. Future research should focus on identifying strategies to support care leaders and exploring the long-term effects of moral distress on healthcare professionals in general and the organizational outcomes associated with moral distress. To identify methods to alleviate moral distress and improve ethical competence in older adult care, an extended investigation of care leaders’ experiences of moral distress should be part of further research.
